# Potts model for haplotype associations

**DOI:** 10.1186/1471-2156-6-S1-S64

**Published:** 2005-12-30

**Authors:** Elena V Moltchanova, Janne Pitkäniemi, Laura Haapala

**Affiliations:** 1 International Institute for Applied System Analysis (IIASA), A-2361 Laxenburg, Austria; 2 University of Helsinki, School of Medicine, Department of Public Health, Mannerheimintie 172, 00014 University of Helsinki, Finland; 3 National Public Health Institute, Department of Health Promotion and Epidemiology, Mannerheimintie 166, 00300 Helsinki, Finland

## Abstract

Bayesian spatial modeling has become important in disease mapping and has also been suggested as a useful tool in genetic fine mapping. We have implemented the Potts model and applied it to the Genetic Analysis Workshop 14 (GAW14) simulated data. Because the "answers" were known we have analyzed latent phenotype *P*1-related observed phenotypes affection status (genetically determined) and i (random) in the Danacaa population replicate 2. Analysis of the microsatellite/single-nucleotide polymorphism-based haplotypes at chromosomes 1 and 3 failed to identify multiple clusters of haplotype effects. However, the analysis of separately simulated data with postulated differences in the effects of the two clusters has yielded clear estimated division into the two clusters, demonstrating the correctness of the algorithm. Although we could not clearly identify the disease-related and the non-associated groups of haplotypes, results of both GAW14 and our own simulation encourage us to improve the efficiency and sensitivity of the estimation algorithm and to further compare the proposed method with more traditional methods.

## Background

Bayesian smoothing methods, which during the recent decade have been widely used in the field of spatial epidemiology [1-3], have recently been proposed as a tool for haplotype effect estimation in fine mapping [4,5]. Such spatial modeling of haplotype effects is based upon some measure of "similarity" between haplotypes and upon the belief that similar haplotypes would affect the phenotype in the same way. Cluster models allow us to go a step further and to group haplotypes according to the magnitude of such an effect. In this paper we report the results of implementing the Potts model [3] using the reversible jump Markov chain Monte Carlo (rjMCMC) technique. The model was applied to the Genetic Analysis Workshop 14 (GAW14) simulated microsatellite and single-nucleotide polymorphism (SNP) data on the Danacaa population replicate 2 on chromosomes 1 and 3 for the affection status (genetically determined) and phenotype i (randomly selected). The results of the Potts model are compared to the results of a more traditional conditional auto-regressive (CAR) model [1].

## Methods

Let *Y*_*i *_denote the observed dichotomous phenotypes of a sample of *i *= 1, ..., *I *subjects, where *Y*_*i *_= 1 if the subject *i *is a case and *Y*_*i *_= 0 otherwise. Suppose also that for every subject a haplotype *H*_*i *_= *h*_1*i*_, *h*_2*i *_is determined with certainty and that some further information on the subjects such as age and sex is available in the form of a covariate matrix **X**. Assuming a standard logistic regression penetrance model we have:



where *δ*_*h *_is the effect of the haplotype *h *on the probability of exhibiting the studied phenotype and *γ *is the vector of effects of the individual covariates.

Here  and the corresponding inverse function . The CAR model of the form



has been considered (e.g., Thomas et al. [4,5]) in the context of genetics. Here *w*_*hk *_are the elements of a symmetrical weight matrix with a null diagonal. In genetic modeling of haplotype effects weights can be defined as the number of alleles shared by a pair of haplotypes or some other more complex genetically based measure. This Bayesian spatial model (BYM), described in detail in Besag et al. [1] has been widely used, especially in epidemiology. More recently several clustering models have been proposed, among them the Potts model [3] of the form



where *z*_*h *_denotes the "cluster" to which the haplotype *h *belongs and  is a relative risk parameter common to all the haplotypes assigned to a particular cluster *z*. In the absence of additional covariates **X **the likelihood may be written down as:



For the identification purposes we have set: *δ*_1 _<*δ*_2 _< ... <*δ*_*k*_, where *k *is the number of clusters. In this setup the number of clusters *k *as well as the allocation vector *z *also have to be estimated. In the Potts model formulation the elements of *z *are modelled jointly conditional on the number of clusters, *k*:



where  and  are the number of like-labeled neighbor pairs in the configuration *z *and an additive normalizing constant, respectively.

For large *k *the normalizing constant cannot be evaluated analytically and has to be precomputed. Because of this we need to take *ψ *to have a discrete distribution uniform on the values {0, 0.1,..., *ψ*_*max*_}. Also, here we assume the prior on the number of components to be uniform on the values {1, 2, ..., *k*_*max*_}, but a more informative prior such as Poisson may be employed instead (e.g., to indicate preference for the smaller number of clusters).

In order to set up a full Bayesian model, we also need to assign the prior to the parameters *δ *and *τ*. If we assign to each component of the vector *δ *a vague normal distribution with mean 0 and precision (i.e., inverse variance) 0.01^2^, the joint prior for *δ *may be written as



Commonly MCMC methods are used in fitting Bayesian models. However because the number of clusters *k *is unknown here, a special dimension-switching move is required along with the usual fixed dimension moves. Therefore a rjMCMC algorithm has been used here [6].

## Results

### Danacaa, D03S0126–D03S0127

Due to the computational complexity of the model we restricted our analysis to Danacaa population using replicate 2. Because the "answers" were known, we chose to use phenotypes affection status and i.

**Figure 1:**
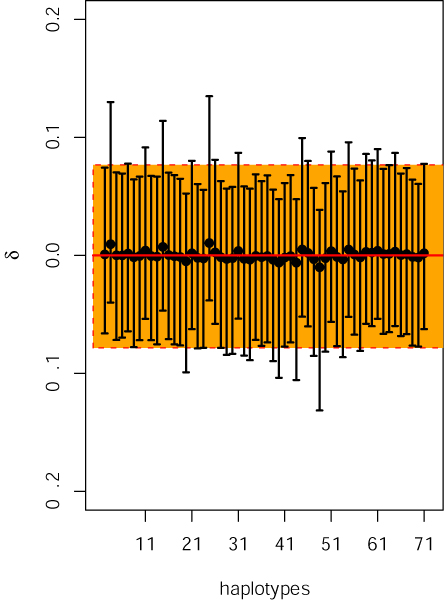
**Danacaa, replicate 2, D03S0126–D03S0127**. Estimated posterior means and 95% confidence intervals for the effects of individual haplotypes for both CAR (56 individual haplotype effects) and Potts models (a single cluster effect, represented by the orange area).

Affection status is determined through disease loci D1 and D2 in complex manner. However, D1 determines phenotypes b and a and D2 e, f, g. Because D1 is located at chromosome 1 and D2 at chromosome 3. For the analysis of D1-associated haplotypes we chose microsatellite markers D01S023–D01S024 and the corresponding SNP was B01TT0561. Correspondingly, for the D2 we chose D03S126 and D03S127 and SNP B03T3067. Haplotypes were constructed using neighboring markers for both microsatellite and SNP data using PEDPHASE program [7]. As a comparison we analyzed trait i, which has no genetic determinants involved using the same haplotypes. The rjMCMC algorithm has been implemented in R [8], the CAR model used for comparison was run on BUGS [9]. In each case 100,000 iterations were run, of which 50,000 were discarded as a burn-in stage. The convergence was assessed visually by graphical examination of the envelopes and traces of the chains. The haplotypes having a common allele at either of the markers were regarded as neighbors. As an example we present the results of the analysis of the microsatellite markers of the Danacaa population replicate 2, area D03S0126–D03S0127. There were a total of 7 * 8 = 56 different possible haplotypes present in the sample. The average prevalence of the affected-trait (Kofendrerd Personality Disorder) in the Danacaa population was 0. The results of both the CAR model and the Potts model, which had suggested *k *= 1 as the most likely number of clusters (*p*(*k *= 1) = 0.9999), are shown in Figure 1.

### Additionally simulated data

**Figure 2:**
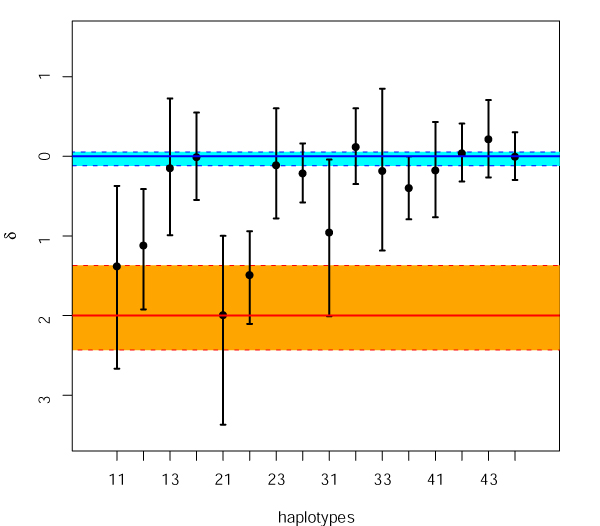
**Separately simulated data modelled on Danacaa replicate 2, D01S023–D01S024**. Estimated posterior means and 95% confidence intervals for the effects of individual haplotypes for both CAR (16 individual haplotype effects) and Potts models (correctly estimated two clusters represented by the orange area for the haplotypes 11, 12, 21, and 22, and by the blue area for the rest). The true simulated values of *δ *= (-2, 0) are shown by solid red and blue lines respectively.

In order to ensure the proper functioning of the algorithm, we have also used additionally simulated data. It was modeled on the haplotypes observed in the Danacaa population, replicate 2 D01S023–D01S024. We took the simulated haplotypes of the Danacaa population and have simulated phenotypes for the subjects based on the haplotypes they possessed. This was done by dividing haplotypes into two clusters corresponding to the pre-defined phenotype effects (*δ *= (-2, 0)). The haplotypes 11, 12, 21, and 22 were set to belong to the cluster with effect *δ*_1 _= -2 and the rest to the cluster with *δ*_2 _= 0. The results of the estimation using rjMCMC and the comparison with CAR model has proved satisfactory and are illustrated in Figure 2 and in Table 1. The posterior probability was estimated *p*(*k *= 2) = 0.98 and the grouping of haplotypes into the two clusters was identified correctly.

## Discussion

Fine mapping is gaining importance as a tool in the search of the genetic basis of complex traits, while knowledge of the patterns of the human linkage disequilibrium is increasing. We have implemented the Bayesian spatial approach proposed in [5]. Our results for the GAW14 Danacaa population replicate 2 with microsatellite marker haplotypes in the neighborhood of disease locus D1 failed to identify any haplotype grouping. However, when applied to the data simulated for the same haplotypes with effects set up into two groups with the effects *δ *= (-2, 0) the model has correctly sorted haplotypes into the two clusters and estimated the effects. It can be concluded therefore that in the case of Danacaa population the model has not proved sensitive enough to detect the effect on the provided sample size. The BYM model [1], which has been widely used for example in the field of spatial epidemiology for over a decade, has been used for comparison. It has also failed to produce evidence of clustering, since the resulting 95% confidence intervals for all the haplotypes are overlapping.

There are certain technical difficulties in estimating the Potts model, one of which is the evaluation of the normalizing constant. We have used the thermodynamic integration approach as proposed by Green and Richardson [3] in conjunction with the Simpson's Rule. Other difficulties lie in constructing the efficient sampling algorithm. The Poisson model used by Green and Richardson [3] in conjunction with gamma priors on the effects leads to certain 'nice' results, but the high incidence of some phenotypes (e.g., e and f) does not allow the natural binomial distribution to be approximated by Poisson. Therefore, we had to deal with a rather unwieldy logit transformation. We plan to improve the algorithm further by searching for a better sampling distribution so as to provide better mixing and faster convergence. We found both the sampling schema and the complexity of the phenotype very challenging and because of the complex model used we have ignored ascertainment. Therefore the estimated haplotype effects reflect only the sample at hand and not the prevalence in the base population.

The similarity matrix of the haplotypes in this study is based on the number of alleles identical by state, but from the genetics point of view it would be more informative to use identity by descent information that can be obtained from other genetic computer software programs such as PEDPHASE [7]. In the future we plan to use more simulations in order to gain better understanding of the statistical properties of the Potts model in its applications to genetic fine mapping of complex diseases. Some comparison between SNPs and microsatellite markers will also be considered, provided the time required to estimate model parameters can be reduced.

## Conclusion

The aim of this article was to test the usefulness of the Potts approach in the genetic analysis. Unfortunately, the results of were not encouraging because neither the Potts nor the comparable BYM model found any haplotype grouping. However, as noted in the discussion, we believe that the approach may work in certain situations. More investigation is needed to determine the conditions under which the proposed approach may prove useful.

## Abbreviations

BYM: Bayesian spatial model

CAR: Conditional auto-regressive

GAW14: Genetic Analysis Workshop 14

rjMCMC: Reversible jump Markov chain Monte Carlo

SNP: Single-nucleotide polymorphism

## Figures and Tables

**Table 1:** Estimation results for the simulated data. The table presents the result of Bayesian estimation for the simulated data for the Potts and CAR models.

	BYM			Potts		
			
Haplotype *h*	*δ*_*h*_-mean	*δ*_*h*_-95% CI	'true' *z*_*h*_	*z*_*h*_-mean	*δ*_*z*_-mean	*δ*_*z*_-95% CI
11	-1.3835	(-2.6663, -0.3718)	1.0000	1.0358	-1.8666	(-2.4328, -1.3697)
12	-1.1221	(-1.9223, -0.4116)	1.0000	1.0341	-1.8666	(-2.4328, -1.3697)
13	-0.1517	(-0.9910, 0.7257)	2.0000	1.9842	-0.0313	(-0.1198, 0.0554)
14	-0.0130	(-0.5484, 0.5487)	2.0000	1.9962	-0.0313	(-0.1198, 0.0554)
21	-1.9963	(-3.3693, -0.9992)	1.0000	1.0008	-1.8666	(-2.4328, -1.3697)
22	-1.4933	(-2.1043, -0.9405)	1.0000	1.0068	-1.8666	(-2.4328, -1.3697)
23	-0.1148	(-0.7801, 0.6017)	2.0000	1.9962	-0.0313	(-0.1198, 0.0554)
24	-0.2165	(-0.5794, 0.1610)	2.0000	1.9945	-0.0313	(-0.1198, 0.0554)
31	-0.9589	(-2.0073, -0.0414)	2.0000	1.3716	-0.0313	(-0.1198, 0.0554)
32	0.1150	(-0.3489, 0.6017)	2.0000	1.9968	-0.0313	(-0.1198, 0.0554)
33	-0.1863	(-1.1841, 0.8477)	2.0000	1.9532	-0.0313	(-0.1198, 0.0554)
34	-0.4010	(-0.7919, -0.0027)	2.0000	1.9929	-0.0313	(-0.1198, 0.0554)
41	-0.1790	(-0.7660, 0.4302)	2.0000	1.9958	-0.0313	(-0.1198, 0.0554)
42	0.0357	(-0.3165, 0.4102)	2.0000	1.9973	-0.0313	(-0.1198, 0.0554)
43	0.2117	(-0.2653, 0.7057)	2.0000	1.9972	-0.0313	(-0.1198, 0.0554)
44	-0.0071	(-0.2966, 0.3009)	2.0000	1.9969	-0.0313	(-0.1198, 0.0554)
